# Source Attribution of Antibiotic Resistance Genes in Estuarine Aquaculture: A Machine Learning Approach

**DOI:** 10.3390/antibiotics13010107

**Published:** 2024-01-22

**Authors:** Helena Sofia Salgueiro, Ana Cristina Ferreira, Ana Sofia Ribeiro Duarte, Ana Botelho

**Affiliations:** 1 Faculty of Veterinary Medicine, University of Lisbon, 1300-477 Lisbon, Portugal; 2 National Institute for Agrarian and Veterinary Research (INIAV IP), Av. da República, Quinta do Marquês, 2780-157 Oeiras, Portugal; cristina.ferreira@iniav.pt; 3 BioISI—Instituto de Biosistemas e Ciências Integrativas, Faculdade de Ciências, Universidade de Lisboa, 1749-016 Lisbon, Portugal; 4 National Food Institute, Technical University of Denmark, Kemitorvet 204, 2800 Kongens Lyngby, Denmark

**Keywords:** aquaculture sediments, antimicrobial resistance, resistomes, source attribution, machine learning

## Abstract

Aquaculture located in urban river estuaries, where other anthropogenic activities may occur, has an impact on and may be affected by the environment where they are inserted, namely by the exchange of antimicrobial resistance genes. The latter may ultimately, through the food chain, represent a source of resistance genes to the human resistome. In an exploratory study of the presence of resistance genes in aquaculture sediments located in urban river estuaries, two machine learning models were applied to predict the source of 34 resistome observations in the aquaculture sediments of oysters and gilt-head sea bream, located in the estuaries of the Sado and Lima Rivers and in the Aveiro Lagoon, as well as in the sediments of the Tejo River estuary, where Japanese clams and mussels are collected. The first model included all 34 resistomes, amounting to 53 different antimicrobial resistance genes used as source predictors. The most important antimicrobial genes for source attribution were tetracycline resistance genes *tet*(*51*) and *tet*(*L*); aminoglycoside resistance gene *aadA6*; beta-lactam resistance gene *blaBRO-2*; and amphenicol resistance gene *cmx_1*. The second model included only oyster sediment resistomes, amounting to 30 antimicrobial resistance genes as predictors. The most important antimicrobial genes for source attribution were the aminoglycoside resistance gene *aadA6*, followed by the tetracycline genes *tet*(*L*) and *tet*(*33*). This exploratory study provides the first information about antimicrobial resistance genes in intensive and semi-intensive aquaculture in Portugal, helping to recognize the importance of environmental control to maintain the integrity and the sustainability of aquaculture farms.

## 1. Introduction

Antibiotic residues can accumulate in the environment due to several anthropogenic activities, leading to selective pressure on bacteria from the environmental microbiome [1,2]. The occurrence of antibiotic resistance in the environment is a major concern due to the spread of resistant bacteria and resistance genes and the associated human health risks [3]. In fact, several international efforts are in place to incentivize the monitoring of antimicrobial resistance in the environment, e.g., the regular monitoring of antimicrobial resistance in the outlets of urban wastewater treatment plants in all agglomerations above 100,000 persons will become mandatory in the EU [4]. The presence of antimicrobial resistance genes (ARG) has been described in bacteria from several environments, namely in marine and aquaculture sediments and soil [5]. A recent meta-analysis study of 460 published articles revealed that the aquaculture sector is a reservoir of resistance to antibiotics with therapeutic potential [1], and other recent scientific reviews [6,7] highlight the importance of the aquatic environment in the transmission of antimicrobial resistance.

Most of the studies about the spread of antibiotic resistance have been focused on acquired resistance [8]. However, in nature—specifically, in water and soil—antibiotic resistance is frequent and mostly intrinsic [9]. This is relevant in terms of antibiotic resistance ecology as it may create charity mechanisms that favor the acquisition of resistance by some community members [10], and some of the genes that are intrinsic in some species may become acquired in others [11]. Shotgun metagenomics sequencing approaches have contributed to the analysis of the span of antimicrobial resistance genes (ARGs) in a sample, irrespective of their intrinsic or acquired character, designated as the resistome [12].

The development and critical systematic assessment of genomic-based source attribution models of antimicrobial resistance (AMR) determinants enable the investigation of resistance gene transmission between several habitats and hosts. The analysis of genetic determinants of resistance derived from metagenomics sequencing, together with the appropriate epidemiological data, are valuable to determine the origin of AMR contamination in aquatic environments [13] and to predict possible contamination from, e.g., different animal reservoirs [14].

Aquaculture located in river estuaries, near industrial sites, farms and urban activities, is subjected to diverse anthropogenic influences and the likelihood of contamination with antimicrobial resistance is therefore high [15]. Sulfonamide resistance genes *sul1* and *sul2*, trimethoprim resistance gene *dfrA1* and class 1 integron *intI1* have persisted for six years in aquaculture sediments in Finland [16] and also in South Korea, China and Japan [17,18,19].

Most Portuguese aquaculture facilities focus on the production of marine fish and bivalve molluscs and operate essentially in estuaries and coastal lagoons, in intensive or semi-intensive systems. This is the case for the semi-intensive production of clams and oysters, which corresponded to over three quarters of the total mollusc production in Portugal in 2018 [20]. Oyster aquaculture has been growing in recent years in Portugal, and only a few studies have been performed to assess its impact on the surrounding ecosystem. A recent study has focused on the selection of antibiotic resistance by metals in riverine bacterial communities in Portugal [21].

In the comprehensive field of global health, machine learning tools have broadly intervened in morbidity and mortality risk assessment and the prediction of certain diseases’ progression [22,23], infectious disease surveillance [24,25,26] and the improvement of health policy and planning [27,28]. For this reason, the interest in applying these tools to antimicrobial resistance genomic data has intensified over the past few years, not only reflecting the exponential increase in genomic AMR data available but also the increasing global awareness of the public health threat posed by AMR. In the current study, the machine learning random forest algorithm was applied to predict the relative attribution of antimicrobial resistance genes (ARGs) to different aquatic environments, based on 34 resistomes of sediments of aquaculture and of estuarine sediments where bivalves are captured.

## 2. Results

### 2.1. RF1—Source Attribution to Aquatic Environment Using Resistomes of Mussel, Gilt-Head Sea Bream and Oyster Aquaculture Sediments

After fitting the first random forest model with the train set, we obtained its performance metrics. The RF1 training model selected nine variables to use randomly at each split of the trees (mtry) and reached accuracy of 0.99. The confusion matrix of the Out-of-Bag (OOB) predictions showed that the model classified all samples correctly, resulting in an OOB error of 0%.

Similarly to training, a summary of the model’s performance was obtained, after making predictions with the 25% hold-out set. The model incorrectly classified one sample, mistaking the Aveiro Lagoon for the Sado River, reaching an accuracy value of 0.95. The Mathews Correlation Coefficient (MCC) reached a value of 0.93.

Overall, the performance results demonstrated that the model was capable of correctly attributing different classes of aquatic environment to observations based on the abundance levels of various AMR determinants.

**Figure 1:**
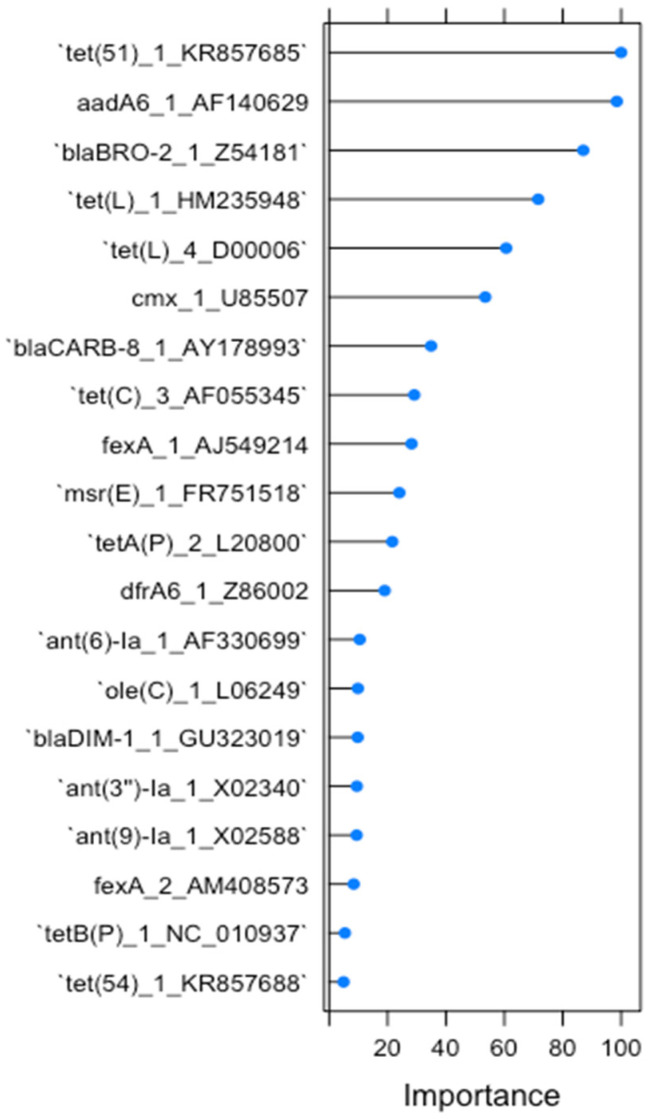
The 20 most important AMR determinants for classification in RF1.

The built-in variable importance measure of RF resulted in the ranking of the twenty most important variables for the classification of RF1, which can be seen in Figure 1. Only six of these twenty variables were demonstrated to have high importance (importance value above 50). We focused our descriptive analysis of gene relative abundance on these six antimicrobial gene clusters, which included the tetracycline resistance genes *tet*(*51*), *tet*(*L*)*_1* and *tet*(*L*)*_4*; the aminoglycoside resistance gene *aadA6*; the beta-lactam resistance gene *blaBRO-2*; and the amphenicol resistance gene *cmx_1*. Among these genes, *tet*(*51*) reached an importance level of 100 and *aadA6* an importance level of 99, thus contributing together to the most prediction accuracy in RF1.

**Figure 2:**
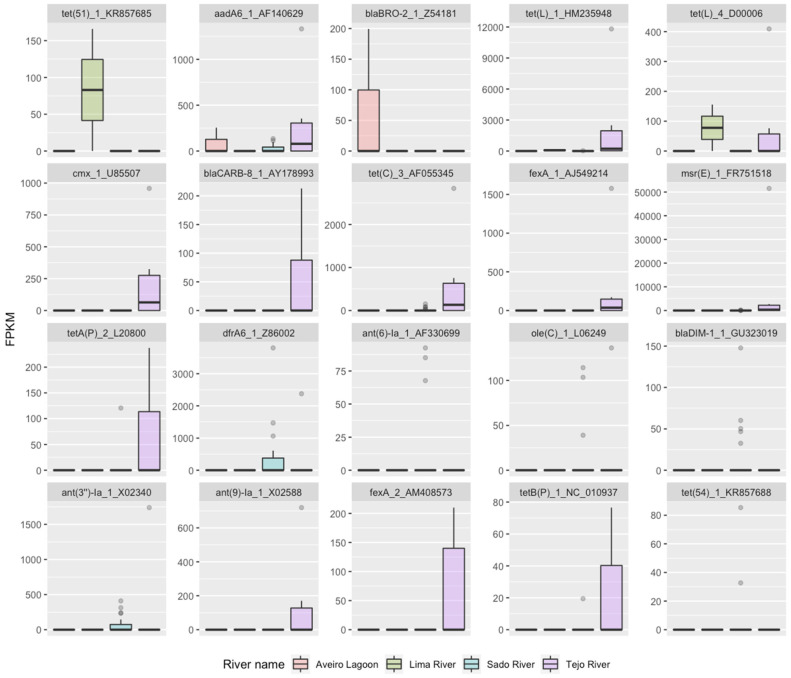
Distribution of abundance in FPKM values of the 20 most important AMR determinants for classification of aquatic environment in RF1.

The distribution of the relative abundance of the twenty most important variables in RF1 considering the environment of the aquaculture of mussels, gilt-head sea bream and oysters (Figure 2) demonstrated that most genes were present in only one aquatic environment.

Among the six genes with the highest importance, *tet*(*51*) was only present in the Lima estuary (oyster production), *blaBRO-2* was only present in the Aveiro Lagoon (oyster production), and *cmx_1* was only present in the Tejo estuary (clam and mussel collection), while *tet*(*L*) was present in two environments (Lima and Tejo estuaries), and *aadA6* was present in three (Aveiro Lagoon and Sado and Tejo estuaries). Ten out of the twenty genes with the top importance in RF1 were detected mostly or exclusively in the Tejo River and six in the Sado estuary (gilt-head bream and oyster aquaculture). In the Aveiro Lagoon and Lima estuary, only two out of the twenty genes were detected in each, *aadA6* and *blaBRO-2* in the first case and *tet*(*51*) and *tet*(*L*) in the latter.

### 2.2. RF2—Source Attribution to Aquatic Environment Using Resistomes of Oyster Aquaculture Sediments

The RF2 model training performance showed accuracy of 0.83. The model selected four random variables at each split of the trees. The confusion matrix of the OOB predictions demonstrated that the model incorrectly classified seven of the fifty-seven observations, resulting in an OOB error of 15.56%. Six of the misclassifications were between the Lima and Sado estuaries, and another between the Lima estuary and Aveiro Lagoon.

Regarding prediction performance with the 25% hold-out set, the RF2 model had accuracy of 0.83 and an MCC of 0.77. It incorrectly predicted two resistomes from the Sado estuary, classifying one as the Aveiro Lagoon and the other as the Lima estuary.

Compared to RF1, the RF2 performance results were not as positive, mostly because the MCC was significantly lower, indicating that the RF2 model might not be as reliable as RF1.

**Figure 3:**
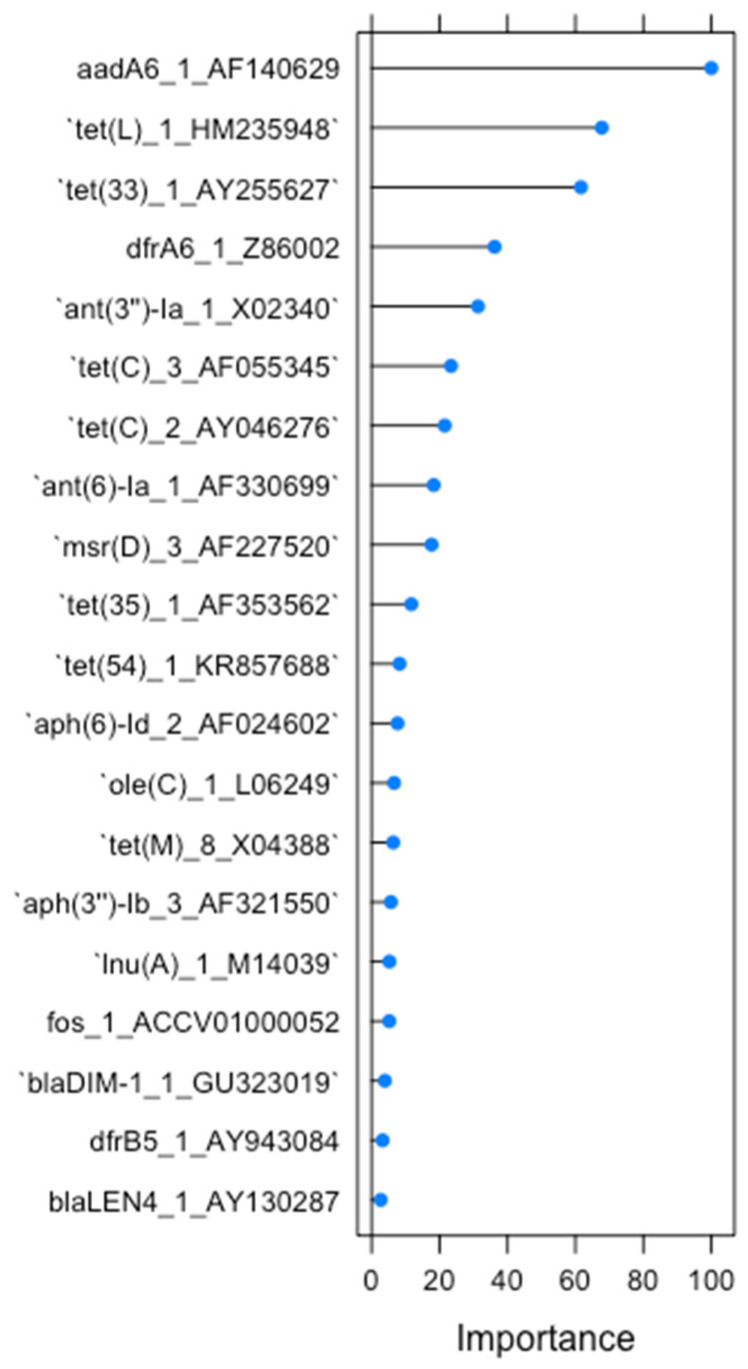
The 20 most important AMR determinants for classification in RF2.

As explained previously for RF1, we were able to assess the twenty most important variables for RF2, shown in Figure 3. For RF2, only three antimicrobial resistance genes had importance above 50, including the aminoglycoside gene *aadA6* and the tetracycline genes *tet*(*L*) and *tet*(*33*). Gene *aadA6* reached an importance value of 100, being the most important gene for classification, while the following two genes, *tet*(*L*) and *tet*(*33*), had an importance level of 67 and 61, respectively.

**Figure 4:**
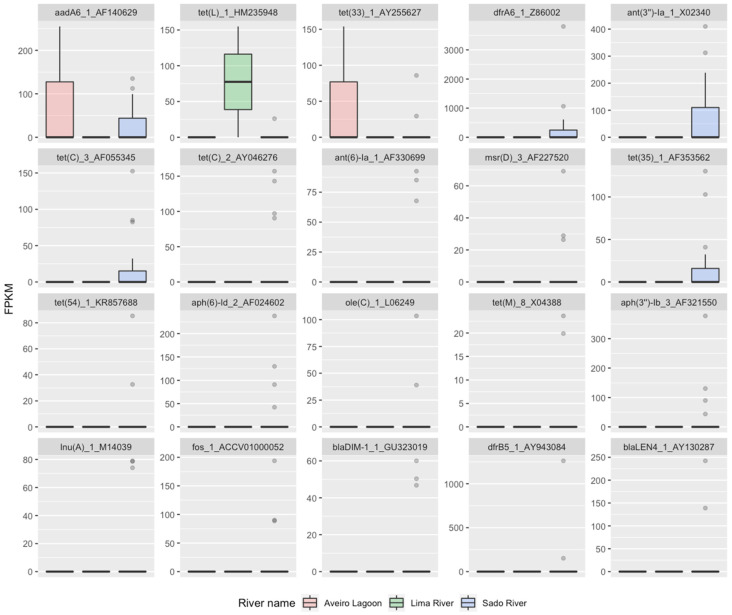
Distribution of abundance in FPKM values of the 20 most important genes for classification of aquatic environment in RF2.

We inspected the distribution of abundance of the twenty most important variables in RF2 (Figure 4). The three genes with the highest importance were the ones present in more than one environment. Genes *aadA6* and *tet*(*33*) were detected in the Aveiro Lagoon and Sado estuary and *tet*(*L*) was detected in the Sado and Lima estuaries. The remaining seventeen of the twenty most important resistance genes in RF2 were only detected in the Sado estuary, mostly sporadically, with a few exceptions that were often detected, including *dfrA6*, *ant*(*3″*)*-Ia*, *tet*(*C*) and *tet*(*35*). Among all twenty variables, only *tet*(*L*) was present in the Lima estuary, and only *aadA6* and *tet*(*33*) were present in the Aveiro Lagoon.

## 3. Discussion

The health sector has increasingly benefited from the function of machine learning algorithms over the years, particularly for the study of AMR, due to the facilitated access to AMR genomic datasets [29]. In this exploratory study, we applied a random forest algorithm to a metagenome-based AMR dataset (resistome) with the purpose of linking the sources of AMR genes to different aquaculture systems located in the estuaries of rivers Sado, Tejo and Lima and in the Aveiro Lagoon.

Random forest has been demonstrated to be one of the most accurate classification algorithms for genomic data analysis, being applied in an increasing number of studies [30,31]. Despite being known for its high accuracy and ability to learn from extensive data, RF models have some limitations, which can lead, in specific scenarios, to suboptimal performance. The main limitation to the use of RF is its tendency to overfit, which is a critical problem when training machine learning models. Overfitting occurs when a model learns the training data too well, in such a way that it memorizes its noise and idiosyncrasies, instead of learning the underlying patterns. There are many causes of overfitting, the most common ones being an insufficient sample size and incorrect model tuning. A model too tuned for the training set (trees excessively deep, or too many trees) will lead to a complex and specific model for these samples only [32]. The present dataset is restricted to only 34 observations, which is a small sample size considering the purpose of fitting an RF model. A small number of observations limits the learning content during the training process, which implies that the model will not be able to make accurate predictions with new observations; thus, it cannot be generalized to new datasets [30,31]. Moreover, these data are exceedingly unbalanced, both in terms of river location and aquaculture species. From the 34 observations, 70% were from oysters and 23 came from Rio Sado, 6 from Rio Tejo and only 2 from Rio Lima and 3 from the Aveiro Lagoon. Unbalanced data is one of the greatest challenges when it comes to RF models. Although we upsampled the data to guarantee the proportion of the same class samples, this was done by replicating the minority samples, and some studies suggest that oversampling very unbalanced data can also lead to overfitting, due to the similarity between the same class samples and low diversity of patterns [29].

Here, we applied two different models, RF1 and RF2, which differed in the number of aquaculture species included, and consequently in the number of samples.

Comparing both models’ performance, RF1 demonstrated superior results compared to RF2. The high accuracy of the model RF1, considering the limited sample size and its unbalanced nature, most likely resulted from overfitting. In order to produce a reliable model, the number of samples collected from each river should be significantly higher and the distribution of samples among the different habitats should be balanced.

In this study, we assessed the 20 most important antimicrobial resistance genes for the model predictions and their abundance among river sites. For both models, the abundance of these genes was barely distributed among all rivers, showing most of the genes only present in the Sado estuary. This may be related to the fact that there is a greater number of samples from this river.

However, the genes with a higher level of importance (*tet*(*51*), *aadA6*, *blaBRO-2*, *tet*(*L*)*_1*, *tet*(*L*)*_4* and *cmx_1* for RF1; *aadA6*, *tet*(*L*)*_1* and *tet*(*33*) for RF2 appear to be those present in a wider variety of estuaries. This indicates that the model learned from the diversity of patterns and based its predictions on the different abundance of genes between aquatic environments.

The acquired genes *tet*(*L*) and *tet*(*33*) confer resistance to tetracycline and code for energy-dependent membrane-associated proteins that export tetracycline out of the cell [33]. Tetracyclines have become one of the most widely used classes of antibiotics in agriculture and aquaculture, due to their broad antimicrobial spectrum, oral availability and low cost. Extensive use over the past seven decades has selected for the expansion of tetracycline resistance in environmental microorganisms [34,35]. As a result of extensive anthropogenic use, tetracycline resistance is now widespread and has frequently been found in fresh water aquaculture [36,37,38].

No discriminatory AMR pattern was observed within the three main locations of oyster aquaculture (Sado and Lima estuaries and Aveiro lagoon), located several kms apart. Based on model RF2, a resistance gene fingerprint of Portuguese oyster estuarine aquaculture could include tetracycline (*tet*(*L*) and *tet*(*33*)) and aminoglycoside (*aadA6*) genes. Previously, Silva and colleagues [15] also reported resistance to tetracycline classes, applying a comparative genomics approach to the same metagenome samples.

Most of the specimens of these oyster aquaculture systems are for export and are controlled in terms of temperature, pH and metal ions. Thus, unless a sporadic event of wastewater treatment plants (WWTPs) or fabric discharge occurs, the resistome of the sediments in these production sites is expected to be somewhat constant. The regular monitoring of AMR patterns in the precise surrounding environments of such aquaculture could help to detect and trace back contamination events in time and space.

For example, in Sado’s estuary, where most of the samples were collected, two regions could be identified: one surrounded by industrial activity (namely paper factories; tomato, milk and fertilizer producers; and two wastewater facilities) and another surrounded by small pig farms. In this second region, residues of enrofloxacin, a fluoroquinolone antibiotic used for the treatment or prophylaxis in pigs [39], were detected in the flesh of the gilt-head bream from the aquaculture (results not shown), suggesting the occurrence of spillover contamination from the pig production environment to the aquaculture environment.

Furthermore, considering model RF1 and its 20 most important AMR genes, Tejo’s estuary presented eleven of these genes, which is remarkable when compared with the other environments, where there was a predominance of only one gene. This distinct resistome might be explained by the non-controlled environment in an area with strong anthropogenic influence, where bivalves are collected by fishermen in a non-licensed place.

At first glance, RF2 seems to have a less confusing dataset for the model, as it predicts estuaries using the patterns of just one species, oysters, distributed among three estuaries. However, the results demonstrate that, even though the number of samples from mussels and gilt-head sea bream is small and they are present in only one estuary each, they help to increase the overall performance. We interpret that the presence of these samples increases the model performance because, since mussels and bream are only present in one estuary, they possibly affect the resistomes of the different aquatic environments considered in the model, thus increasing the sensitivity in prediction.

This study demonstrated that, despite the limitations in terms of sample size and balance, the resistome found in the sediment of aquaculture environments is possibly influenced by the species under production and probably consequently the different production practices. The differences encountered in the resistomes were sufficient to inform a random forest model that could successfully predict the aquatic environment of origin of a sediment sample. It also highlighted a clear difference in resistome composition between controlled aquaculture production sites and non-controlled environments where the unlicensed collection of bivalves occurs. In order to improve the model’s validity and exclude possible confounding effects, larger datasets and relevant epidemiological data (e.g., proximity to farms or wastewater treatment plants), respectively, are needed in the future.

## 4. Materials and Methods

### 4.1. Sample Collection and DNA Extraction

Portuguese oyster aquaculture in the Sado and Lima estuaries and in the Aveiro Lagoon and gilt-head bream aquaculture in the Sado estuary were selected due to their importance and volume of production. In the Tejo estuary, a large water body surrounded by seven municipalities and industries with effluent discharges, Japanese clams and mussels are captured, and, due to this wild anthropogenic activity, this region was also selected.

Between November 2018 and July 2019, 24 samples of oyster (*Crassostrea angulata*) aquaculture sediments were collected in three estuarine regions, near urban centers: the Lima estuary, near Viana do Castelo city in the north of Portugal (two samples); the Aveiro Lagoon, near Aveiro city in the center (three samples); and the Sado estuary, near Setúbal city in the south (19 samples). Four sediments of gilt-head bream (*Sparus aurata*) aquaculture were also collected in Sado’s estuary. In the Tejo River estuary, Japanese clams (*Ruditapes philippinarum*) and mussels (*Mytilus* spp.) are freely collected for human consumption, and, from there, six sediments were collected. All 34 sediment samples were stored at −20 °C until processing for total DNA extraction using a Qiagen PowerSoil Pro extraction kit, ID 47016 (Germany), according to the manufacturer’s instructions. The DNA concentration and quality were determined using a Qubit 4.0 Fluorometer (Thermo Fisher Scientific, Waltham, MA 02451,USA). High-quality DNA samples (≥500 ng high molecular weight in a concentration of ≥20 ng µL^−1^) in at least a 25 µL volume were used for library construction.

### 4.2. Metagenomic Sequencing, Reference Databases and Bioinformatic Analysis

Metagenomic sequencing was performed at CeGaT (GmbH Tuebingen, Germany) with the NovaSeq 6000 platform (Illumina, Inc., San Diego, CA 92122 USA), as well as whole genome sequencing, followed by the preparation of the sequencing libraries (Illumina DNA (M) Tagmentation Library Prep kit), with a read length of 2 × 100 bp, resulting in an output of about 20 billion bases (10 M clusters) per sample. Demultiplexing of the sequencing reads was performed with Illumina bcl2fastq (2.20) and adapters were trimmed with Skewer (version 0.2.2). The quality of the FASTQ files was analyzed with FastQC (version 0.11.5-cegat) and the data were delivered as trimmed FASTQ files.

### 4.3. Machine-Learning Based Source-Attribution

**Figure 5:**
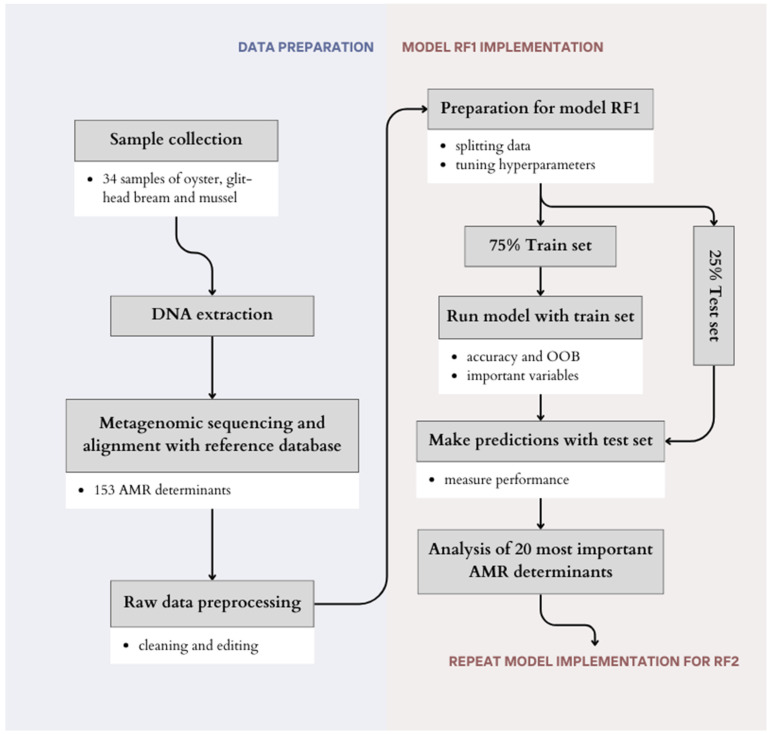
Study design.

All analyses conducted in this study were performed in R Studio v.1.3.1093, and the full study design is presented in Figure 5.

The supervised machine learning algorithm random forest (RF) was applied to predict the class of a resistome observation regarding the aquaculture environment. Random forest is a tree-based supervised machine learning algorithm that is highly adaptive and is able to account for correlations and interactions among explanatory variables, also called features [40]. RF can be a collection of hundreds or thousands of decision trees. Each tree uses a bootstrap sample of the original data, and binary splits recursively partition the tree based on the most popular classification, pushing the samples from a parent node to its two daughter nodes, so that the homogeneity in the daughter nodes is improved [30]. The model uses a random number of features in each split to increase the accuracy and randomization. When this collection of trees is generated, a final vote is cast based on the classification of every tree [41]. Prediction for each observation is based on the proportion of votes given to each class across all trees, in the form of relative probabilities, from which the model produces a final classification based on the most likely class—the “crisp class” [42].

Two models were applied in this study, named RF1 and RF2, using the R package *caret* v.6.0-93 [43]. The two models used the same data; however, RF1 included all samples and RF2 included only oyster samples.

In terms of data preprocessing, all zero and near-zero variance features were removed, and highly correlated features were assessed and eliminated using the *findCorrelation* function of *caret* [43]. This function creates a correlation matrix and returns the pairwise correlations that are above a given cutoff and the features to be removed in order to reduce the level of multicorrelation in the data. A balanced proportion of samples of different classes was assured by upsampling the data. The approach of upsampling with replacement was used, where all original data are left intact and additional samples are added to the minority classes, with replacement [44].

In both models, tuning of the model’s hyperparameters was performed, which consisted of (a) establishing 200 as the number of decision trees to be generated (*ntree*), (b) selecting 10-fold cross-validation repeated 5 times as a resampling method and (c) defining an interval of *mtry* values (number of variables used at each tree split). Each model then selected the *mtry* value that provided the best performance.

Both datasets were randomly split into a train set and a hold-out set, corresponding to 75% and 25% of the original data, respectively. Each model was trained with the 75% train set and using the *train* function of R package *caret* v6.0-93 [43], after which the algorithm presented the final model’s characteristics, such as the *mtry* and model performance assessed based on accuracy and Out-of-Bag (OOB) error.

In RF, accuracy is defined as the number of correct classifications divided by the number of samples. The built-in accuracy metric of the *train* function ranges from 0 to 1 and is calculated using the k-fold cross-validation method, where the model is trained on k-1 folds and uses the remaining fold to test predictions. This process is repeated n times, after which the accuracy is then calculated as the average proportion of correctly classified instances over all folds [45].

The built-in OOB error in RF is described as the fraction of incorrect classifications over the number of out-of-bag samples. When each bootstrap sample is selected from the training data, the observations that are left out are called out-of-bag samples, which are extremely useful to estimate the generalization error and variable importance. Each OOB sample is passed down the tree to produce an estimated prediction error for the sample [46].

The final model was fit to the 25% hold-set to make predictions, using the *predict* function of the caret package. A confusion matrix was composed based on crisp-class classifications, and the prediction performance was assessed using the accuracy and Mathews Correlation Coefficient (MCC). The MCC is a statistical metric used for model evaluation, calculated based on the occurrence of true positives (TP), false positives (FP), true negatives (TN) and false negatives (FN) [47]. This metric ranges from −1 to 1, where 1 indicates perfect agreement between the prediction and observation, −1 indicates total disagreement and 0 is expected for a prediction no better than random. The MCC was used because it has been shown to be more reliable than other performance metrics, such as the RF accuracy values [48].

After evaluating the model performance, the twenty most important variables for classification were analyzed. The RF importance measures help to understand the significance of each variable in the model [49]. For this task, the *varImp* function of the *caret* R package was used [43]. In each decision tree, every time a variable splits a node, the reduction in impurity (Gini impurity) in the two daughter nodes is calculated. The Gini impurity is a measure of the randomness in the split of the decision tree [50]. The *varImp* function calculates the importance score of each variable by averaging this reduction in impurity caused by the variable over all decision trees in the RF model [43]. The more a variable contributes to the reduction in impurity, the higher its importance score. The scores are then scaled such that they range from 0 to 100, to make it easier to compare the importance scores of different variables.

The distribution of abundance of the top twenty genes with the highest importance was plotted for each class of observations, to visualize differences in abundance between classes. Details of each generated model are described below.

#### 4.3.1. RF1—Source Attribution to Aquatic Environment Using Mussel, Gilt-Head Bream and Oyster Resistomes

The first random forest included all data available. These data consisted of 34 resistome observations from oysters, mussels and gilt-head bream, distributed among 4 aquatic environments situated in Portugal (Sado, Tejo and Lima estuaries, as well as Aveiro Lagoon). Among all resistomes, 153 different antimicrobial resistance genes were detected. After upsampling and the removal of features with near-zero variance and highly correlated features, the data consisted of 92 observations, 23 of each environment and 53 features (antimicrobial resistance genes). After data splitting, the train set consisted of 52 observations and the hold-out set of 20 observations. The model was fit using an interval from 4 to 12 as the *mtry*, in order to include, with some margin, the square root of the number of features (rule of thumb of *mtry* values).

#### 4.3.2. RF2—Source Attribution to Aquatic Environment Using Oyster Resistomes

To exclude possible species-related resistome variations, a single species was selected to run a second random forest (RF2). Due to the larger representation of different aquatic environments among oyster samples, a second model was performed including only observations of oyster resistomes. Oyster samples were collected in 3 of the 4 environments; thus, RF2 had 3 output classes (Sado and Lima estuaries and Aveiro Lagoon). Originally with 24 observations, after upsampling and the removal of predictor features with zero variance, the data consisted of 57 observations, distributed among 3 aquatic environments, and 30 predictor antimicrobial resistance genes. After splitting, the train set consisted of 45 observations and the hold-out set of 12 observations. Maintaining the same criteria used in RF1, the interval of values used as *mtry* ranged from 2 to 10.

## Data Availability

The data presented in this study are openly available at https://www.researchgate.net/publication/365620017_Database_of_Metgenomes_of_Sediments_from_Estuarine_Aquaculture_Farms_in_Portugal-AquaRAM_Project_Collection accessed on 11 November 2022.
